# How Internet Contracts Impact Research: Content Analysis of Terms of Service on Consumer Product Websites

**DOI:** 10.2196/23579

**Published:** 2020-12-02

**Authors:** Caitlin Weiger, Katherine C Smith, Joanna E Cohen, Mark Dredze, Meghan Bridgid Moran

**Affiliations:** 1 Department of Health, Behavior & Society Johns Hopkins Bloomberg School of Public Health Baltimore, MD United States; 2 Institute for Global Tobacco Control Department of Health, Behavior & Society Johns Hopkins Bloomberg School of Public Health Baltimore, MD United States; 3 Department of Computer Science Johns Hopkins University Baltimore, MD United States

**Keywords:** marketing, contracts, internet, jurisprudence, ethics

## Abstract

**Background:**

Companies use brand websites as a promotional tool to engage consumers on the web, which can increase product use. Given that some products are harmful to the health of consumers, it is important for marketing associated with these products to be subject to public health surveillance. However, terms of service (TOS) governing the use of brand website content may impede such important research.

**Objective:**

The aim of this study is to explore the TOS for brand websites with public health significance to assess possible legal and ethical challenges for conducting research on consumer product websites.

**Methods:**

Using Statista, we purposefully constructed a sample of 15 leading American tobacco, alcohol, psychiatric pharmaceutical, fast-food, and gun brands that have associated websites. We developed and implemented a structured coding system for the TOS on these websites and coded for the presence versus absence of different types of restriction that might impact the ability to conduct research.

**Results:**

All TOS stated that by accessing the website, users agreed to abide by the TOS (15/15, 100%). A total of 11 out of 15 (73%) websites had age restrictions in their TOS. All alcohol brand websites (5/15, 33%) required users to enter their age or date of birth before viewing website content. Both websites for tobacco brands (2/15, 13%) further required that users register and verify their age and identity to access any website content and agree that they use tobacco products. Only one website (1/15, 7%) allowed users to display, download, copy, distribute, and translate the website content as long as it was for personal and not commercial use. A total of 33% (5/15) of TOS unconditionally prohibited or put substantial restrictions on all of these activities and/or failed to specify if they were allowed or prohibited. Moreover, 87% (13/15) of TOS indicated that website access could be restricted at any time. A total of 73% (11/15) of websites specified that violating TOS could result in deleting user content from the website, revoking access by having the user’s Internet Protocol address blocked, terminating log-in credentials, or enforcing legal action resulting in civil or criminal penalties.

**Conclusions:**

TOS create complications for public health surveillance related to e-marketing on brand websites. Recent court opinions have reduced the risk of federal criminal charges for violating TOS on public websites, but this risk remains unclear for private websites. The public health community needs to establish standards to guide and protect researchers from the possibility of legal repercussions related to such efforts.

## Introduction

### Background

A growing proportion of morbidity and mortality globally can be attributed to the commercialization of products that are harmful to health. Products such as tobacco, alcohol, fast food, and firearms account for an increasing proportion of preventable deaths, a trend referred to as the *industrial epidemic* [1]. For example, globally, there were 2.8 million deaths associated with alcohol consumption and over 8.1 million deaths associated with tobacco consumption in 2017 [2]. Dietary risk factors (including diets high in sodium, low in vegetables, low in fruit, low in whole grains, low in nuts and seeds, and low in seafood omega-3) caused 10.9 million deaths in 2017 [2]. In the United States alone, in 2015, firearms were responsible for over 36,000 deaths [3] and caused 612,000 deaths between 1999 and 2017 [4].

The industries that manufacture these products continue to rely heavily on marketing to attract and retain consumers to maintain and grow their profit margins; these actions contribute to the overall burden of disease caused by their products [5]. Although estimates of the marketing expenditures of the firearms industry are not currently available, tobacco, alcohol, and fast-food companies spend billions, often tens of billions of dollars, on advertising each year [6-9]. There is substantial evidence to suggest that exposure to tobacco, alcohol, food, and firearm marketing increases the desire to use, intentions to use, and/or actual use of advertised products [8,10-27]. Children and adolescents are particularly susceptible to advertising and promotional efforts, given their developmental stage, and are more likely to adopt attitudes and preferences congruent with advertisements after exposure [28]. In instances where products can have detrimental effects on health, advertising becomes a major public health concern.

The research literature on marketing practices for potentially harmful products at the point of sale, on television, and in print media is longstanding and robust [8,13,21,23,24,26,29-31]. The placement of advertising and promotional efforts is, however, increasingly dominated by the web-based domain [32], which provides the possibility of reaching more than 4.5 billion people in the world with internet access [33]. Companies are transferring traditional marketing principles to the internet as *e-marketing* by using websites, social media, and web-based marketplaces to increase connections with potential customers.

Advertising on brand websites has already started to attract the attention of researchers. Research on alcohol brand websites, for example, has documented strategies that utilize youth culture, including computer games, competitions, downloadable content, sponsored parties, fashion shows, and sporting events [34]. Tobacco internet marketing has also been highlighted as a surveillance priority [35]. Descriptive research has found that tobacco brand websites offer sweepstakes; event promotions; video advertising; and contests involving music creation, lyric writing, creative design, and other arts to engage users on the web [36-39]. Food and beverage websites have been found to contain *gamified* content that is designed specifically for children and often includes games that highlight branded content (adver-games), branded downloadable content, viral content, and unlimited commercials [40-42]. However, no research has assessed the marketing content of gun websites, although recent research characterizing firearm advertising on Twitter and YouTube has been published [43]. Both firearm manufacturers and social media influencers on these platforms promoted guns for recreational and military use, and used patriotic and law enforcement themes. Importantly, this research also revealed that social media posts, particularly YouTube posts, often connect viewers to firearm manufacturer websites [43]. Previous research has established that brand websites are filled with promotions and advertising strategies meant to interactively engage users in a way that is not possible with print media or even television commercials.

The use of e-marketing is also potentially problematic because it is more difficult for regulatory authorities to monitor and regulate web-based space, resulting in less oversight [44,45]. The enforcement guidelines of the Food and Drug Administration (FDA) for the 2010 Tobacco Control Act states that it will conduct surveillance of internet promotions to ensure compliance with advertising and promotion restrictions, although it is unclear how this is carried out in light of terms of service (TOS) and authorization restrictions [46]. It is possible that companies are taking advantage of the less regulated web-based world to employ more aggressive advertising strategies that are already restricted in other media. For instance, researchers have found that some content on UK alcohol brand websites may violate UK broadcasting codes because of the likelihood that it would appeal to underage consumers [47]. There is a need for regulatory authorities to ensure that there is no gap in oversight of web-based commercial spaces. Surveillance has been referred to as the foundation of public health [48], and monitoring and curtailing advertising of potentially harmful and intrinsically harmful products is an important aspect of public health practice.

E-marketing, although clearly a source of concern for public health, poses challenges for researchers. Websites are the property of corporations that draft TOS that can legally shape how site users are allowed to engage with content. The *Law Dictionary* defines terms and conditions (abbreviated TOS) as “Special and general arrangement, rule, requirements, standards, etc. Forming integral parts of a contract or agreement” [49]. Website TOS typically take on 2 forms: click-wrap agreements and browse-wrap agreements. Click-wrap agreements require that the user clicks a box or otherwise actively indicates that he or she assents to the TOS before he or she is able to access website content [50]. Browse-wrap agreements, on the other hand, do not require the user to actively agree to the TOS. Instead, the TOS are located somewhere on the web page, which is usually accessible by a link at the bottom of the page. Courts have ruled inconsistently regarding whether TOS legally constitute notice, which is required for a contract to be binding, although click-wrap agreements have been upheld more routinely than browse-wrap agreements [51-55]. The legally ambiguous nature of TOS makes it unclear if the prohibitions often outlined in these terms are legally binding, and research has shown that they are rarely read or understood [56-58].

Frischmann and Selinger [59] maintained that the length and complexity of TOS are intentional design features. They described TOS contracts as “techno-social tools for engineering human beings to behave automatically, like simple machines.” TOS contracts are *take-it-or-leave-it* contracts, where users can either accept terms or not access the website. This means that website users gain nothing by reading many pages of legal jargon, as they have no power to change the terms of the contract, and are able to reduce the cognitive cost of the encounter by ignoring TOS [59]. Frischmann and Selinger [59] state that website designers function as *choice architects* by shaping the choices that users are presented with and that TOS are subject to this design influence as much as website content is. As the choice regarding TOS is to accept the TOS or not use the website, unthinking, constant acceptance becomes the norm as people become *programmed* to agree [59]. Legal scholar Margaret Jane Radin additionally raises concerns regarding *boilerplate* *creep*, where private entities, such as corporations, gradually replace law from public spaces such as governments and social institutions with their own *law* that is designed to benefit the private entity rather than the public good [60]. These concerns that TOS are intentionally designed to deflect website user attention and at the same time benefit private entities underlies the importance of better understanding the content of TOS contracts and how they may influence research.

When TOS are actually read, it becomes clear that many activities necessary to conduct research are often restricted. Preliminary exploration found TOS on brand websites that prohibit users, for example, from downloading (eg, saving copies of content for future reference) and sharing material, tasks necessary to conduct research. The terms of entry into web-based spaces designed by corporations are more explicit and seem to hold the potential for greater enforcement in the web-based domain as compared with conditions for entry into retail spaces in the physical environment. The uncertain legal nature of TOS may also impact the comfort level of the researchers who study web-based spaces, and some researchers have expressed fear that this uncertainty might have chilling effects on research [61].

### Goal of This Study

Little attention has been paid to establishing appropriate norms for entering web-based spaces for the purpose of understanding the space in and of itself. Although a recent publication [62] discusses the content of TOS on social media websites, focusing on both automated and manual data scraping of user content, conducting research on consumer product websites is somewhat different. Content analyses would not necessitate scraping and do not document user content the way social media research does. TOS on consumer product websites require the same kind of consideration that TOS on social media websites have received. The Association of Computing Machinery, a professional organization for computing scientists, has already started to discuss if it is ethical to violate TOS during the process of data collection of publicly available information [63,64]. The association has even appealed to the court in an amicus curiae briefing, arguing that researchers should be legally allowed to conduct research on public web-based spaces [65]. This issue also needs to be considered by public health researchers.

Currently, researchers are leaving themselves potentially vulnerable to legal issues by conducting research that might violate the TOS of websites. Given the public health importance of monitoring product marketing in web-based spaces, in this study, we describe the types of activities that are and are not allowed by TOS across multiple product types and what types of restrictions are being put on website access. We also consider how this might differ by product type and discuss implications of restrictions included in TOS for research and the ethical and legal ramifications of not adhering to TOS, given recent litigation.

## Methods

### Setting and Sample

We searched Statista, the “statistics portal for market data, market research, and market studies” [66] for the top branded consumer products in 5 domains relevant to public health: tobacco (cigarettes), alcohol (beer and distilled spirits), firearms, prescription psychiatric drugs, and fast food. In addition to the *industrial epidemic* products described earlier, we included prescription drug websites because of the heavy use of direct-to-consumer marketing [67], a practice only allowed in the United States and New Zealand [68], and the association between exposure to this marketing and subsequent desire to use such drugs [17-19]. We choose to focus on psychiatric drugs due to the high prevalence of use among American adults [69]. To identify the top brands in these domains, we searched Statista for the top brands in the United States by market share for each product. When data on market shares by brand were not available, we searched for top brands by revenue from sales. We selected the highest ranked brands that listed the law governing their TOS as a state within the United States and that had an official website. Official websites were defined as websites created by the actual company rather than a fan group or a specific retailer. We restricted our study to US companies that listed a location within the United States as governing their TOS because we wanted to control for some variance in TOS by the country that the company operated in, as regional laws and requirements may differ by country.

For cigarette brands, the 3 US market leaders, Marlboro, Newport, and Camel, were all American companies with official websites created by the company. Marlboro and Newport were selected for the sample. Camel was excluded because both Camel and Newport were owned by R. J. Reynolds Tobacco Company and had the same TOS. Data on US market leaders for beer brands showed that Bud Light and Coors Light occupied the largest portion of the beer market and were retained for the sample. Statista did not have data on distilled spirits as a general category; however, it had existing data on the top whiskey brands by market share, with Jack Daniels, Crown Royal, Fireball, and Jim Beam being the market leaders. The British company Diageo owns Crown Royal and was excluded, whereas Fireball, Jack Daniels, and Jim Beam were retained as US-owned brands. Although Statista did not have data on the top market leaders in the firearms industry, it did provide data on the estimated global revenue for firearms made for the US market, which was used as a proxy for establishing the leading companies. The top 3 firearm manufacturers—Remington, Smith and Wesson, and Sturm Ruger—are all US companies with official websites and were retained for this sample. Similarly, the top companies producing psychiatric drugs were also determined using Statista data on revenue from top-selling psychiatric drugs in the United States. The top brand, Lyrica, was retained for the sample. The second brand, Vyvanse, stated in its TOS that it is governed by British, rather than American law, and the website was excluded from the sample for this reason. Invega Sustenna was retained as this brand had an official website, and the jurisdiction listed in its TOS was within the United States. Revenue from sales was also used to determine the top fast-food brands. McDonalds, Starbucks, and Subway were retained for this sample as the jurisdiction listed in their TOS was within the United States, and all the brands had official websites (Table 1). In total, 15 websites were included in the sample.

**Table 1:** Acquisition of website sampling using data from Statista.

Product type and metric used/Top brands listed		Webcite reference for the brands selected	Date of last TOS^a^ update (as of July 2018)	Date of last TOS update (as of July 2020)
**Cigarettes, market share of the leading US cigarette brands in 2016 (%)**				
	Marlboro, 41	[70]	May 2014	May 2014
	Newport, 13	[70]	August 17, 2017	August 17, 2017
	Camel, 8	—^b^	—	—
**Beer, domestic beer market share of the leading brands in the United States in 2017 (%)**				
	Bud Light, 18	[71]	No date provided	January 1, 2020
	Coors Light, 10	[72]	May 22, 2018	January 1, 2020
**Whiskey, US market share of the leading whiskey brands in** **2017 through 2018** **, based on dollar sales (%)**				
	Jack Daniels, 13	[73]	March 15, 2018	March 15, 2018
	Crown Royal, 12	—	—	—
	Fireball, 8	[74]	June 21, 2017	June 21, 2017
	Jim Beam, 8	[75]	October 29, 2008	January 31, 2020
**Firearms, estimated global revenue made annually for the US civilian market (million US $)**				
	Remington Outdoor, 939	[76]	April 1, 2017	No date provided
	Smith and Wesson, 552	[77]	April 1, 2017	June 1, 2020
	Sturm Ruger, 551	[78]	September 1, 2010	September 1, 2010
**Psychiatric drugs, selected top psychiatric drugs’ US revenue in 2016 (million US $)**				
	Lyrica, 4.4	[79]	No date provided	No date provided
	Vyvanse, 3.1	—	—	—
	Invega Sustenna, 1.3	[80]	November 4, 2016	November 4, 2016
**Fast food, leading quick service restaurants in the United States in 2016, based on retail sales (billion US $)**				
	McDonalds, 36.4	[81]	March 13, 2017	March 13, 2017
	Starbucks, 15.8	[82]	October 27, 2017	October 2019
	Subway, 14.0	[83]	June 14, 2018	January 1, 2020

^a^TOS: terms of service.

^b^Not included in the sample.

Official websites for each brand were located by searching the brand name with the word *website* in Google. Each website was opened, and restrictions on website access, such as pop-up windows and registration pages, were documented. The TOS were located, typically by scrolling to the bottom of the website homepage and looking for a link called *terms of service* or some other equivalent. TOS from each website were copied and pasted into Microsoft Word. All TOS were saved to Webcite (with the exception of the Marlboro website TOS, which blocked Webcite from saving its content) and were also downloaded and saved as PDFs.

### Analysis

All TOS were downloaded and coded in Microsoft Word (Word version 16.34) employing line-by-line open coding. After reading the TOS and conducting an initial round of coding for major themes, codes were discussed with the study team at multiple meetings, and codes were added and refined per group discussion (Textbox 1). Codes were developed based on how specific aspects of TOS could impact research. The primary coder (CW) has considerable experience with both qualitative and quantitative coding. After the primary coder completed coding, 2 additional coders (KS and MM) independently reviewed and confirmed all codes. Discrepancies were discussed and resolved during email exchanges between the coders and the broader research team. The proportion of websites employing each code was calculated in Microsoft Excel (Excel version 16.37), and differences and themes by product type were qualitatively assessed because of the small sample size.

**Codes that emerged from the qualitative process and corresponding examples.**

Age restrictions

- ≥21 years, legal age of product consumption, ≥18 years of age or ≥age of majority, ≥13 years with parental consent, ≥13 years, or no age restrictions

Other access restrictions

- Users must be a user of our product to access this website, and/or users must agree to receive promotional materials

Accepting TOS (terms of service)

- By accessing this website, users agree to abide by the TOS (use of all capital letters were sometimes used to emphasize this point)

TOS can change at any time

- Users acknowledge that TOS can change at any time, users will be notified of changes to TOS, or users are expected to check TOS for any changes during each website visit

Restrictions on sharing account information

- It is the user’s responsibility to keep log-in credentials confidential

User information accuracy

- All information provided by the user must be accurate

Prohibited and allowable user actions with website material

- A series of codes indicating when or if copying, displaying, distributing, downloading, transmitting, translating, and republishing is prohibited or allowed

Applicable local laws

- Users agree to abide by applicable local laws while using this website

Choice of law

- TOS are interpreted and governed by the laws in, for example, North Carolina

Restrict user access

- The company can restrict user access to this website for any reason at any time

Allowable use

- Commercial, personal, private, noncommercial, etc

Actions taken if user violates TOS

- Having the user’s internet protocol address blocked, taking legal action against the user, and/or deleting user content from the website

## Results

### Access and Restrictions

All tobacco (2/2, 100%) and alcohol (5/5, 100%) websites presented with a pop-up window or a registration page when users first enter the website. Both tobacco websites had registration windows, whereas all 5 alcohol websites had pop-up windows. Pop-ups on alcohol websites only required the user to input a date of birth or confirm that they were aged ≥21 years, with no verification process. Two alcohol websites also asked for geographical location. All but 3 websites (12/15, 80%)—2 firearm websites and 1 pharmaceutical website—explicitly stated an age requirement for user access (Table 2). Both tobacco websites additionally required that registrants be tobacco users, with 1 tobacco website also requiring that users sign up for a mailing list and be willing to receive promotional material. No other websites had additional restrictions apart from age.

**Table 2:** Number of websites with age restriction on website access.

Product type	Age ≥21 years	Legal age of product consumption	Age ≥18 years	Age ≥13 years with parental consent if under 18 years or the legal age of majority	Age ≥13 years	No age restrictions
Tobacco (n=2), TOS^a^ number, n (%)	2 (100)	0 (0)	0 (0)	0 (0)	0 (0)	0 (0)
Alcohol (n=5), TOS number, n (%)	2 (40)	3 (60)	0 (0)	0 (0)	0 (0)	0 (0)
Pharmaceuticals (n=2), TOS number, n (%)	0 (0)	0 (0)	1 (50)	0 (0)	0 (0)	1 (50)
Fast food (n=3), TOS number, n (%)	0 (0)	0 (0)	0 (0)	3 (100)	0 (0)	0 (0)
Firearms (n=3), TOS number, n (%)	0 (0)	0 (0)	0 (0)	0 (0)	1 (33)	2 (67)

^a^TOS: terms of service.

The TOS for 10 websites (10/15, 67%; 2 tobacco, 4 alcohol, 1 pharmaceutical, 2 fast food, and 1 firearm) held users responsible for keeping their log-in credentials (should they create an account) private, and the TOS for 6 websites (6/15, 40%) specified that information used to create an account must be accurate (2 tobacco, 2 alcohol, and 2 food). The Newport TOS, for instance, states, “You must sign-up online to create an account to access and use the Site. You agree not to use any false, inaccurate, or misleading information when signing up for your accounts.” Newport is also the only website in the sample that specifies that they independently verify that registrants are aged ≥21 years.

All website TOS (15/15,100%) had language stating that accessing the website required users to accept the TOS, and all but one pharmaceutical company (14/15, 93%) said that the TOS could change at any time and that users may or may not be notified of this fact (Table 3).

**Table 3:** Accepting terms of service and the possibility of changing terms by accessing websites.

Product type	By accessing the website, users agree to the TOS^a^	TOS can change at any time	The user is responsible for checking the TOS for changes and is bound by the changes	Website will post announcements when the TOS change and users are bound by the changes	Used all capitalization to emphasize that by accessing the website, the user agrees to the TOS
Tobacco (n=2), TOS number, n (%)	2 (100)	2 (100)	1 (50)	1 (50)	0 (0)
Alcohol (n=5), TOS number, n (%)	5 (100)	5 (100)	4 (80)	1 (20)	3 (60)
Pharmaceuticals (n=2), TOS number, n (%)	2 (100)	1 (50)	1 (50)	0 (0)	0 (0)
Fast food (n=3), TOS number, n (%)	3 (100)	3 (100)	2 (67)	1 (33)	1 (33)
Firearms (n=3), TOS number, n (%)	3 (100)	3 (100)	3 (100)	0 (0)	2 (67)

^a^TOS: terms of service.

### Local Laws, Jurisdiction, and Restricting Access

Language requiring users to comply with applicable local laws in addition to the terms stated in the TOS was common (2 tobacco, 4 alcohol, 1 pharmaceutical, 2 fast food, and 3 firearms; 12/15, 80%). All TOS (15/15, 100%) also specified a state within the United States whose laws would govern and interpret the TOS and serve as a location for any future litigation. Virginia, North Carolina, Missouri, Illinois, Kentucky, New York, New Jersey, Washington, Connecticut, and Massachusetts (10 states) were listed for the 15-website sample used in this study.

With only 2 exceptions (1 pharmaceutical and 1 alcohol; 13/15, 87%), all websites stated that they could revoke a user’s access at any time, without providing a justification. A total of 73% (11/15) TOS listed specific consequences for breaking or violating the TOS, including having the user’s internet protocol address blocked so they could no longer access the website; pursuing legal action against the user, resulting in civil or criminal penalties; terminating log-in credentials; and deleting user content from the website (2 tobacco, 3 alcohol, 2 pharmaceutical, 2 fast food, and 2 firearms).

### Allowable and Prohibited Actions With Website Content

Each TOS further specified what users were and were not allowed to do with the content of the website. Most of the TOS prohibited using website content for commercial purposes, and all but 3 TOS specified that website material could only be used for personal or individual or noncommercial purposes (1 tobacco, 4 alcohol, 2 pharmaceutical, 2 fast food, and 3 firearms; 12/15, 80%).

Website TOS described specific restrictions on how website content could be used (Table 4). A total of 33% (5/15) TOS unconditionally or near unconditionally prohibited or put substantial restrictions on all of these activities (2 tobacco) and/or failed to specify if they were allowed or prohibited (2 alcohol and 1 food). Substantial restrictions, for instance, were found on the Marlboro website, which stated that there might be instances where users are given explicit permission to use website content outside of the site, but that this use could only be for personal noncommercial purposes and only applied when users were given explicit permission on the web page. A total of 73% (11/15) websites prohibited distributing website content or required prior permission to distribute materials. Moreover, 53% (8/15) websites allowed users to engage in at least one activity that exceeded simply viewing of website material (eg, download, copy, or distribute) for personal or individual noncommercial purposes and often explicitly stated that downloaded content must retain all copyrights and trademarks and may only be allowed in limited circumstances (4 alcohol, 1 pharmaceutical, 2 fast food, and 1 firearm). The same set of websites also prohibited users from sharing website content with others. One pharmaceutical company also fell into this category but additionally allowed distribution for noncommercial purposes. Many website TOS did not specify if displaying (4/15, 27%), downloading (5/15, 33%), copying (3/15, 20%), and translating (8/15, 53%) website content was allowed. Only 1 website in the sample (Invega Sustenna) allowed users to display, download, copy, distribute, and translate website content as long as it was for personal and not commercial use.

**Table 4:** Prohibited or allowable use of website content.

Brand name	Display website content	Download website content	Copy website content	Distribute website content	Translate website content
Marlboro	Prohibited	In circumstances where specific sections of the website say you can use website materials offline, it must be for personal noncommercial purposes	Prohibited	Prohibited	Prohibited
Newport	Prohibited	Prohibited (all actions other than viewing are prohibited unless otherwise specified)	Prohibited	Prohibited (all actions other than viewing are prohibited unless otherwise specified)	Prohibited (all actions other than viewing are prohibited unless otherwise specified)
Bud Light	Prohibited without prior permission	Not specified	Prohibited without prior permission	Prohibited without prior permission	Not specified
Coors Light	Not specified	Not specified	Not specified	Prohibited without prior permission	Not specified
Jim Beam	Prohibited without prior permission	Allowed for noncommercial, lawful, and personal use with copyright retained	Prohibited without prior permission	Prohibited without prior permission	Prohibited without prior permission
Jack Daniels	Not specified	Allowable for one copy for personal, noncommercial use with copyright retained	Prohibited for commercial use	Prohibited for commercial use	Prohibited
Fireball	Prohibited without prior permission	Allowable for personal, noncommercial use with copyright retained and no modifications	Not specified	Prohibited without prior permission	Not specified
Lyrica	Prohibited without prior permission	Allowable for noncommercial individual references with copyright retained	Allowable for noncommercial individual references with copyright retained	Prohibited without prior permission	Not specified
Invega Sustenna	Prohibited for commercial use without prior permission	Allowable for personal, noncommercial purposes with copyrights retained	Prohibited for commercial use without prior permission	Allowable for personal, noncommercial purposes with copyrights retained	Prohibited for commercial use without prior permission
McDonalds	Allowable for personal, noncommercial purposes	Not specified	Prohibited for commercial use	Prohibited for commercial use	Prohibited
Starbucks	Not specified	Not specified	Not specified	Prohibited	Not specified
Subway	Prohibited for commercial use	Not specified	Prohibited without prior permission	Prohibited without prior permission	Prohibited without prior permission
Remington Outdoor	Allowable occasionally with an insubstantial portion of the content for noncommercial purposes with copyrights retained and including “Used with permission from Remington”	Prohibited	Prohibited	Allowable occasionally with an insubstantial portion of the content for noncommercial purposes with copyrights retained and including “Used with permission from Remington”	Not specified
Sturm Ruger	Prohibited without prior permission	Allowable for personal and authorized commercial use	Allowable for personal and authorized commercial use	Prohibited without prior permission	Not specified
Smith and Wesson	Not specified	Allowable for personal, noncommercial, and informational use	Prohibited without prior permission	Prohibited	Not specified

## Discussion

### Principal Findings

Our exploration of TOS has revealed several important conclusions and raised some significant insights that we will discuss in the context of recent litigation: (1) research on web-based spaces is complicated by the existence of TOS in that they restrict activities necessary for research; (2) commercial entities are creating spaces on the web and some use TOS to try to restrict access to those spaces; and (3) research on private web-based spaces is ethically justifiable, regardless of whether the activities necessary to conduct this research are allowed under TOS agreements, but this research is legally questionable, so how should researchers proceed? Companies already have the financial power to aggressively market their products both on and off the web; in addition, they also have the power to define what is and is not allowed regarding the use of their website material via TOS [63,84]. This power apparently allows companies to restrict access to websites and restrict specific activities one can do with the website material. Some of the restricted activities are necessary to conduct research, and the existence of TOS calls into question the legality of conducting such research. Researchers, then, may be left feeling unsure of how to conduct necessary monitoring of web-based commercial spaces without risking legal action [61].

TOS are complicated and cloud the legality of research on consumer product websites. It is important for regulatory agencies to have data on websites to make informed marketing regulations. The FDA, for instance, calls on members of the public, including researchers, to report marketing violations in Section 3.1.4 of the *Tobacco Control Act Enforcement Manual* [46]; however, performing basic tasks to document content and marketing strategies on the web is made difficult or impossible if researchers are bound by TOS such as those outlined. It is notable that these TOS potentially restrict many of the activities needed to conduct a basic content analysis (Table 4), a research method that provides details on the content and marketing strategies employed on websites. For instance, researchers need to save and share website content for content coding, analysis, and potentially for later reporting. All activities that are necessary to conduct a content analysis (which we defined as displaying, downloading, copying, distributing, and translating) were only explicitly allowed by one website TOS. Such restrictions were also noted during an evaluation of TOS on social media websites [62]. Website content changes frequently, and not saving content, which typically requires downloading, would likely result in lost data, which would be untenable for effective surveillance or academic analytic processes. Translation is also sometimes necessary for international work assessing websites used in other countries, necessitating both translation and sharing content with others, activities often prohibited by TOS.

Most of the explored TOS also state that the company can restrict access at any time, sometimes for any reason. If a company chooses to do this in the midst of data collection, it could impose a significant barrier. None of the TOS in this sample provided any information regarding an appeals process to contest restricted access. It is possible that some companies might intentionally restrict access if they think a user is accessing the website for research purposes that might reflect badly on the company, using their TOS-stated right to restrict access at any time and/or for any reason as a rationale.

Researchers could try to create a protocol that would follow the TOS for all the commercial websites they were interested in studying. However, attempting this could be difficult, as TOS can vary considerably across websites and include legal jargon [85,86]. Researchers could limit their sample based on websites that allow the required research activities; however, this would likely result in an unrepresentative sample and could exclude websites where TOS tends to prohibit more activities, such as tobacco websites. It is possible that companies employing more problematic marketing tactics might have more restrictive TOS, and those are the companies that are most important to monitor.

In addition, one of the most consistent finding across product type is terms stating that website content can only be used for personal, noncommercial use. It is unclear if research fits in this category. It seems fairly clear that it is not *personal*, as the purpose of research is to disseminate findings to the scientific community and the public. At the same time, it is also clear that public health research is not a commercial pursuit: profit is not the goal of the endeavor. None of the TOS in our small sample and only one in the larger sample of social media sample examined by Fiesler et al [62] explicitly address the use of websites for research purposes; therefore, it remains unclear if research activities are prohibited by corporations or not. Fiesler et al [62] conclude that TOS on consumer websites, such as TOS on social media websites, are “ambiguous and largely devoid of context,” making them difficult to understand and abide by. Arguments using the First Amendment’s protection of free speech have been raised recently in the Supreme Court, and although the court did not consider those arguments, it is possible that they could be called upon if needed [87].

We must then attempt to untangle what companies claim is binding in their TOS, compared with what the US courts have upheld as legally binding during litigation to better understand if researchers are truly bound by the limitations imposed by TOS. The Computer Fraud and Abuse Act (CFAA) is a federal law that prohibits accessing a computer without authorization or in a manner that exceeds authorization, which can be interpreted as a federal prohibition on TOS violations [88]. The recent memorandum opinion published by the US District Court for the District of Columbia in the case of *Sandvig v. Barr* [87] stated that merely violating TOS on public websites does not constitute a breach of the CFAA for exceeding authorized access to a computer and, therefore, cannot trigger federal criminal charges. In this case, researchers and journalists asked the court for clarity in a pre-enforcement challenge if the work they wanted to conduct (analyzing if algorithms on hiring websites discriminate based on race, gender, age, or other attributes by creating fictitious user profiles) would violate the CFAA and trigger criminal charges. The court stated that in cases where any user can access a website, even if users are required to create a username and password for access (ie, the website is public), violating the TOS is not a CFAA violation. Although this is certainly good news for researchers who want to conduct research on public websites, the court clarified that TOS violations may still trigger federal and state civil charges. Such violations would amount to the web-based equivalent of trespassing; however, as long as researchers are not inflicting damage by slowing down website operations, preventing other user access, or tampering with content, it is unclear what damages companies could request compensation for.

Variability in the jurisdictions selected for governing the TOS and litigation may impact what state civil charges companies could bring for researcher trespass. Companies largely control the state where litigation will occur, and different states may have different laws governing what constitutes trespass and the consequences for trespassing. This introduces more uncertainty in terms of what research activities could result in civil charges.

Although the memorandum in *Sandvig v. Barr* offers researchers conducting research on public websites reassurances, the issue of research on private websites remains of questionable legality. In light of recent litigation, it is also possible that companies will restrict access to their websites and create private spaces where research activities continue to be more limited. Tobacco companies already restrict access to their websites and even require authentication of provided information. A researcher may not be able to gain access to view the selected tobacco website content without providing false information to register (eg, falsely identify themselves as a smoker). Doing so would serve as a breach of TOS because of the requirement that registrants be tobacco users and provide only true information. Violating TOS by conducting research activities that require downloading, sharing, and so on, on a private website may also still constitute a violation of the CFAA, which can trigger federal criminal charges. Tobacco websites were the only websites that are considered private in this sample, and these websites prohibited or severely restricted all the activities necessary to conduct a content analysis.

If researchers are convinced of the public health need to surveil private websites but are not able to do so while adhering to TOS, there are potentially incompatible legal and ethical issues to weigh. The ethical issues raised by such surveillance research are limited to the extent that surveillance activities do not seek to analyze interaction between users but rather seek to document the content of the website as it is designed by companies. In other words, it is a commercial entity rather than an individual who is being surveilled. From an ethical perspective, it is arguable that corporate actions do not warrant the same protection as human subjects, with the result that corporations may not be able to claim the right to autonomy from research participation. There is precedence for treatment of commercial organizations differently from individuals in research; company names are often used in academic publication, and there is no existing standard stating that companies should not have identifiable information disclosed.

The legal issues underlying research on websites vary in magnitude depending on whether the website is public, where anyone can create log-in credentials or access website content without a log-in, or private, where websites require authentication of provided information and apply constraints regarding who is allowed to create log-in credentials. The legal issue of research on public websites appears to be more limited. The biggest threat, violation of the CFAA, has been largely removed, and civil charges at the state or federal level seem unlikely when no damage is done to the websites of interest. Research on public websites should be encouraged and expanded to provide the public health community with a better understanding of e-marketing tactics and opportunities to inform marketing regulations that would better protect public health. This is especially important in the present moment when research requiring face-to-face interaction is limited by the ongoing pandemic. The legal issue of research on private websites presents a greater challenge. Even if a court was to eventually rule that violating TOS on private websites for public health research is not a violation of the CFAA, being sued and facing the litigation that follows is time consuming and has the potential to hurt the credibility and reputation of the researcher. Such damage might reduce the likelihood of promotion or achieving tenure if it is costly for the institution to defend the researcher. This is particularly problematic for private tobacco websites, as the tobacco industry has a long history of targeted and aggressive marketing of a product that is intrinsically harmful to users. Although account requirements may ostensibly be to responsibly keep out youth and nonsmokers, it also limits the access of the researchers and makes their access potentially illegal. Further, there is nothing stopping other companies that sell harmful products from putting up similar restrictions.

This study has also revealed the involvement of research institutions and universities as key stakeholders in decisions regarding research involving TOS violations. The expectations for researchers need to be clearly defined and understood in the various relevant offices of the university such that important research can be undertaken in such a way that the institution is comfortable with and can (and will) stand behind the researchers in the case of industry action. Currently, these issues are beyond the scope of what institutional review boards perceive as their purview, leaving researchers with few resources for guidance on questions of both ethics and legal issues [89].

We would conclude that violating TOS to conduct public health research on product websites is not ethically questionable; rather, given the public health significance of e-marketing, we may be ethically bound to conduct such research, regardless of whether it occurs on public or private websites. This conclusion has similarly been reached by the Association for Computing Machinery's Committee on Professional Ethics in their Code of Ethics and Professional Conduct, which states that TOS and other internet regulations should be followed unless there is a “compelling ethical justification to do otherwise” [90]. The code goes on to state that those who violate TOS for ethical or any other reasons “must accept responsibility for that action and for its consequences” [90]. How, then, should researchers proceed with research on private product websites in a way that minimizes or eliminates legal risks? An important question is whether researchers are in any way precluded from TOS restrictions. Beyond this general question, researchers could contact companies and request permission to study their website. Although this option might grant researchers access, it would also put the company on notice that research is being conducted, and there is no great incentive for companies to agree to surveillance. It is critical that the public health community establish standards for such researchers to enable the continuation of this important work in ways that the researchers and institutions involved are protected.

### Limitations

This study explored a small and purposefully selected sample of commercial product website TOS. It is possible that we missed variability in TOS that might be present on other types of websites. We also limited our sample to US companies, and TOS for companies within the United States might be different from the website TOS for companies in other countries, and caution should be used when attempting to generalize these results. The TOS observed during this study were downloaded and analyzed in July 2018. As of July 2020, 7 websites have updated their TOS. A brief review of updated TOS shows that very little has changed in terms of allowable activities with website material. The only exception to this was Jim Beam. Jim Beam added a clause that “You may print or download one copy of a reasonable number of pages of this Website for your own personal, noncommercial use and not for further reproduction, publication, or distribution” and some language allowing app download. We also employed confirmatory coding, rather than blind double-coding, which may have biased the second coder. Given that we were unable to access the content for tobacco websites, we were unable to conduct a content analysis to determine if our hypothesis that websites with more problematic content would be those with stricter TOS was correct.

In addition, none of the coders were lawyers, although lawyers were consulted to ensure that terms were coded appropriately and their significance was understood. As noted by others who have studied TOS and other similar contracts, terms can approach incomprehensibility, even for legal experts [85,86,91]. We discussed our findings and the legal implications of violating TOS for research with a lawyer and 2 law students at the Cyberlaw Clinic at the Harvard Law School, who validated that the legal landscape is continuously changing and it is currently unclear if creating false accounts on private websites (in the context of gaining access to a tobacco website) would be considered a violation of the CFAA. The US government has never charged a researcher for violating TOS in the course of their research, and the recent Sandvig decision certainly reduces the risk of this for research on public websites; however, the risk of violating the CFAA while researching private websites such as tobacco websites remains unclear.

This study was limited to TOS contracts and did not include the evaluation of privacy policies. A privacy policy is a “legal document that discloses some or all of the ways a party gathers, uses, discloses and manages a customer's data” [92]. As this study did not assess privacy policies, it cannot state what kind of surveillance researchers have to agree to when they are attempting to surveil consumer product websites. This is an important area of future exploration and would add to the existing work on privacy policies by Bagley and Brown [52] and Obar and Oeldorf-Hirsch [56].

### Conclusions

E-marketing on brand websites is an important area for public health surveillance to monitor; however, TOS complicate this endeavor by restricting access and prohibiting activities needed to conduct research. Recent court opinions have reduced the risk of federal criminal charges for violating TOS on public websites, but this risk remains unclear for private websites. Researchers already engaged in research on private websites are putting themselves in danger of being sued, and their affiliated institutions may or may not support them in court. It is critical that the public health community establishes standards for conducting research on e-marketing that supports this important public health issue.

## Abbreviations

CFAA: Computer Fraud and Abuse Act
FDA: Food and Drug Administration
TOS: terms of service
